# Comparative Performance of Calcium‐Modification Strategies for Coronary Lesions: A Network Meta‐Analysis of Randomized Trials

**DOI:** 10.1155/cdr/9434123

**Published:** 2026-08-30

**Authors:** Fiorenzo Simonetti, Andreas T. Kristensen, Tobias Lenz, Felix Voll, Moritz von Scheidt, Michael Joner, Tobias Rheude, Hendrik Sager, Erion Xhepa, Adnan Kastrati, Niels Thue Olsen, Salvatore Cassese

**Affiliations:** ^1^ Department of Cardiovascular Diseases, TUM Klinikum Deutsches Herzzentrum, Technical University of Munich, Munich, Germany, tum.de; ^2^ Deutsches Zentrum für Herz-und Kreislauf-Forschung (DZHK) e.V. (German Centre for Cardiovascular Research), Partner Site Munich Heart Alliance, Munich, Germany; ^3^ Department of Advanced Biomedical Sciences, University of Naples Federico II, Naples, Italy, unina.it; ^4^ Department of Cardiology, Copenhagen University Hospital, Rigshospitalet, Copenhagen, Denmark, rigshospitalet.dk

**Keywords:** atherectomy, calcified coronary lesion, endovascular procedures, intravascular imaging, lesion preparation, modified balloon, percutaneous coronary interventions

## Abstract

**Introduction and Objectives:**

Calcified coronary lesions are associated with an increased risk of percutaneous coronary intervention (PCI) failure. Despite the availability of several calcium‐modification strategies and randomized trials evaluating their efficacy, comparative investigations remain scanty. The aim of the present analysis is to assess the comparative performance of calcium‐modification strategies in patients undergoing PCI of calcified coronary lesions.

**Methods:**

We conducted a systematic search for randomized trials evaluating calcium‐modification strategies in patients undergoing PCI. Random‐effects network meta‐analyses were performed. The primary outcome was minimal stent area (MSA). Secondary outcomes included all‐cause death and stent thrombosis.

**Results:**

Twenty‐two trials enrolling 4855 patients were included. Compared to calcium modification with noncompliant balloons, the use of cutting/scoring balloon (mean difference: 0.81 mm^2^, 95% confidence interval: [0.36, 1.27]; *p* = 0.001), super high‐pressure balloon (SHPB, 0.95 mm^2^ [0.18, 1.72]; *p* = 0.016), intravascular lithotripsy (IVL, 0.87 mm^2^ [0.40, 1.34]; *p* < 0.001), rotational atherectomy (RA, 0.90 mm^2^ [0.30, 1.50]; *p* = 0.003), and RA plus cutting balloon (1.24 mm^2^ [0.46, 2.02]; *p* = 0.002) led to larger MSA. According to a network ranking analysis, RA, SHPB, and IVL were possibly the best treatment options in terms of MSA. There were no significant differences in clinical outcomes between calcium‐modification strategies.

**Conclusions:**

Compared with standard noncompliant balloons, RA and balloon‐based strategies improved the mechanical performance of PCI in calcified coronary lesions. However, this did not result in better clinical outcomes.

## 1. Introduction

A growing number of complex percutaneous coronary intervention (PCI) procedures are conducted due to the aging population and rise in comorbidities [1]. Some of these comorbidities, such as diabetes mellitus and chronic kidney disease, are well‐known risk factors for coronary artery calcification. Indeed, severe coronary calcification is found in up to 30% of patients undergoing contemporary PCI, and this proportion is expected to rise in the coming years [2].

Coronary calcification often impedes the advancement of materials (e.g., guidewires, microcatheters, balloons, and stents), adequate balloon dilation, stent delivery, and implantation. This ultimately impairs the efficacy and safety of PCI through multiple mechanisms [3, 4]. The management of calcified coronary lesions represents a major challenge. In recent decades, various devices and techniques have emerged for calcium modification, in addition to standard balloons (semicompliant balloon [SCB] and noncompliant balloon [NCB]). These include balloon‐based strategies such as modified balloons (MBs), namely, scoring balloon (SB) and cutting balloon (CB), super high‐pressure balloon (SHPB), intravascular lithotripsy (IVL), and atheroablative devices, including rotational atherectomy (RA), orbital atherectomy (OA), and excimer laser coronary atherectomy (ELCA) [5].

Several randomized trials have compared calcium‐modification strategies. However, no study has assessed the comparative performance of available devices directly. For this reason, we conducted a network meta‐analysis to provide an overview of intravascular imaging (IVI), angiographic, procedural, and clinical outcomes across available calcium‐modification strategies for patients undergoing PCI of calcified coronary lesions.

## 2. Methods

This systematic review and network meta‐analysis was performed and reported according to the Cochrane Handbook for Systematic Reviews of Interventions and the Preferred Reporting Items for Systematic Reviews and Meta‐Analyses (PRISMA) (Table S1). [6, 7]

### 2.1. Data Source and Search Strategy

Randomized trials were identified by systematic search in MEDLINE, Embase, and recent conference supplements from each database inception to October 31, 2025. The detailed search algorithm is outlined in Table S2. Two investigators (FS and SC) examined the manuscripts on study design, patient characteristics, and clinical outcomes independently, using standardized spreadsheet forms. Any disagreements were resolved by consensus following discussion with a third investigator (AKa). Records that were not relevant were excluded based on screening of the title and abstract. The remaining records were then evaluated at full‐text level. Where multiple publications were derived from the same randomized trial, the study providing the most detailed data was selected. Where data details overlapped, the study with a follow‐up most closely matching the overall median follow‐up time was selected to enhance consistency of follow‐up across the included studies.

### 2.2. Eligibility Criteria

Studies were considered eligible if they met the following criteria: (1) randomized trials, (2) comparison of calcium‐modification strategies in patients undergoing PCI and stenting of a calcified coronary lesion, and (3) availability of imaging and clinical outcomes. We excluded subanalyses of randomized trials in which the original randomization was not performed between calcium‐modification devices and studies comparing different strategies using the same device (e.g., RA with different rotational speeds). No restrictions were applied with regard to sample size, publication language, or definition of calcified lesions. To ensure the most comprehensive and up‐to‐date evidence base, conference abstracts and presentations from major cardiology meetings within the predefined search period were also considered, allowing the inclusion of recently completed randomized trials (Figure S1).

### 2.3. Quality Assessment

The Cochrane Risk‐of‐Bias 2 (RoB 2) tool for randomized studies served to assess the risk of bias for each included trial. A formal transitivity assessment between treatments was performed comparing the distribution of selected effect modifiers across the different included trials [7–9]. The Confidence In Network Meta‐Analysis (CINeMA) framework was used to evaluate confidence in the results [10].

### 2.4. Outcomes

The primary outcome of this analysis was minimal stent area (MSA) after PCI. Secondary outcomes included angiographic post‐PCI diameter stenosis (DS), all‐cause death, myocardial infarction (MI), stent thrombosis (ST), repeat revascularization (defined as target lesion or vessel revascularization), coronary perforation, and procedural success. Detailed definitions of all outcomes, according to each trial, are provided in Table S3.

### 2.5. Statistical Analyses

The mean difference (MD) with 95% confidence interval (95% CI) was used to calculate the differences for the continuous parameters MSA and DS. The remaining outcomes were compared using risk ratios (RRs) with 95% CI. Statistically significant results were indicated by *p* values < 0.05. Both direct and indirect estimates between interventions were generated using a random‐effects model with a frequentist graph‐theoretical approach, with NCB set as the reference treatment. A network plot for the primary outcome served to visualize the network geometry. The consistency between the direct and indirect evidence was assessed using the node‐splitting method, which involves splitting the contributions of each comparison into direct and indirect evidence and assessing the contrast between the two components of the evidence. The *I* ^2^ statistic was used to evaluate heterogeneity, with values ≥ 50% considered significant [11]. *p* score ranked treatments (rankogram) were measured for each outcome on a scale from 0 (*worst*) to 1 (*best*) [12]. Given the peculiar nature of the MSA and procedural success outcomes, for which higher values indicate better performance, the *p* score ranking was inverted for these two analyses (0 = *best* and 1 = *worst*). Publication bias was assessed using funnel plot and Egger′s linear regression tests to quantify asymmetry [13]. Due to the structural similarity, CB and SB were grouped as MB. Sensitivity analyses were conducted for the primary outcome: Firstly, we excluded trials with a high risk of bias in one or more components of the RoB 2. Secondly, we excluded trials with only an angiographic definition of calcified lesions. Sensitivity analyses were also performed for the clinical outcomes according to follow‐up duration (under or above the median) and whether IVI was utilized to define the degree of coronary calcification or to guide the PCI. In addition, to account for the varying follow‐up durations of the included studies, the incidence rate ratio (IRR) with 95% CIs was calculated for all‐cause death, MI, ST, and repeat revascularization. All analyses were performed using the “meta” and “netmeta” packages with R software Version 4.4.1 (R Foundation for Statistical Computing).

## 3. Results

The flow diagram for trial selection is shown in Figure S1. A total of 22 trials [14–35] enrolling 4855 patients were included.

**Figure 1:**
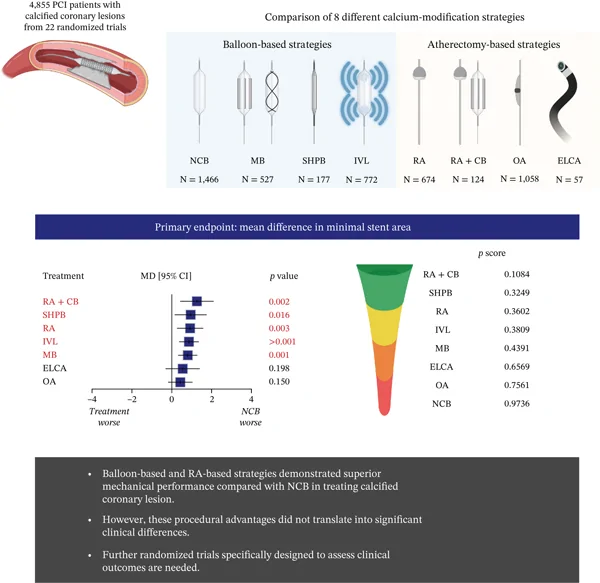
Main findings of the study. The modified balloon category for lesion preparation includes both cutting balloons and scoring balloons. CB, cutting balloon; CI, confidence interval; ELCA, excimer laser coronary angioplasty; IVL, intravascular lithotripsy; MB, modified balloon; MD, mean difference; NCBs, noncompliant balloons; OA, orbital atherectomy; SHPB, super high‐pressure balloon; PCI, percutaneous coronary intervention; RA, rotational atherectomy.

Four out of 22 trials were available as conference presentations [15–18]. A total of eight calcium‐modification modalities were compared: NCB (*n* = 1466), IVL (*n* = 772), MB (*n* = 527), SHPB (*n* = 177), ELCA (*n* = 57), OA (*n* = 1058), RA (*n* = 674), and RA plus CB (*n* = 124, Figure 1). The trial characteristics are provided in Table S4. The sample sizes ranged from 40 to 2005 patients. The majority of the trials were multicenter and showed no ethnic prevalence. Most of the trials were two‐arm, with only one trial having a three‐arm design [36]. Eighteen trials included IVI in their protocol, either to assess coronary calcification at baseline, to guide PCI procedures, or both [14–16, 18, 19, 22–27, 29, 32–34, 36–38]. Of these 18 trials, 8 used optical coherence tomography, while intravascular ultrasonography was used in the remaining trials. Two trials used both modalities [34, 37]. The proportion of patients with severe coronary calcifications was reported in 17 out of 22 trials. Five trials excluded patients with a recent diagnosis of acute coronary syndrome [23, 24, 27, 31, 32]. Overall, the risk of bias was high in three studies due to concerns regarding the selection of reported results [21, 23, 33], in one trial due to issues related to both outcome measurement and the selection of reported results [27], and in four trials for which data were extracted from conference presentations, primarily due to missing information (Figure S2) [15–18]. Results of the CINeMA assessment are presented in Table S5. Overall, comparisons supported exclusively by indirect evidence were rated as having low confidence, mainly because of major concerns in the incoherence domain. The sample‐size weighted median follow‐up was 12 months. Table 1 shows the baseline and procedural characteristics of each trial. The average patient age was 71.6 years, more than two‐thirds were male, and nearly 40% had diabetes mellitus. The majority of treated lesions were located in the left anterior descending artery.

**Table 1:** Baseline and procedural characteristics in the included trials.

Trial	Patients, *n*	Age (mean)	Male (%)	Diabetes mellitus (%)	LAD treated (%)	Severe calcification (%)
BALI [14]	200	74	76.5	31.0	56.5	96.5
BASIL [19]	60	71	66.7	23.3	41.7	100
COPS [33]	87	71	81.6	34.5	58.6	NA
DIRO [32]	100	74	69.0	61.0	69.0	NA
ECLIPSE [34]	2005	70	73.0	43.9	67.3	100.0
Elyamani et al. [31]	30	65	70.0	60.0	66.7	100.0
EXIT‐CALC [25]	40	76	65.0	35.0	52.5	100.0
Han et al. [27]	120	71	65.8	35.0	54.2	NA
ICARE [38]	169	72	81.1	36.5	69.8	71.6
ISAR‐CALC [24]	74	73	85.1	33.8	54.1	98.6
Li et al. [26]	71	76	70.4	73.2	60.5	NA
PREDICTION [15]	170	74	76.0	44.0	NA	76.0
PREPARE CALC [30]	200	75	76.0	33.5	69.5	74.5
ROLLER COASTR [36]	171	71	78.9	49.5	70.2	81.6
ROTA.Shock [29]	61	73	75.4	36.1	39.3	100.0
ROTA‐CUT [23]	60	71	78.3	41.6	56.6	73.3
ROTAXUS [28]	240	71	75.8	27.1	65.8	46.9
Short‐CUT [18]	413	72	75.1	45.1	42.6	NA
SONAR [17]	170	74	77.4	32.0	55.1	85.2
Tang et al. [22]	92	61	68.5	17.4	84.8	100.0
VICTORY [16]	282	71	84.9	27.0	NA	60.8
Zou et al. [21]	40	69	55.0	20.0	87.0	100.0

Abbreviations: ACS, acute coronary syndrome; BALI, Balloon Lithoplasty for Preparation of Severely Calcified Coronary Lesions; BASIL, Balloon Angioplasty versus Shockwave Intravascular Lithotripsy; COPS, Cutting Balloon to Optimize Predilatation for Stent Implantation; DIRO, direct comparison of RA vs. OA for calcified lesions guided by OCT; ECLIPSE, Evaluation of Treatment Strategies for Severe CaLcIfic Coronary Arteries: Orbital Atherectomy vs. Conventional Angioplasty Technique Prior to Implantation of Drug‐Eluting StEnts; EXIT‐CALC, EXpansion of stents after Intravascular lithotripsy versus conventional predilatation in CALCified coronary arteries; ICARE, Intravascular Lithotripsy in Comparison to Rotational Atherectomy: An Evaluation by OFDI; ISAR‐CALC, ComparIson of Strategies to PrepAre SeveRely CALCified Coronary Lesions; LAD, left anterior descending artery; LCx, left circumflex artery; LM, left main artery; NA, not available; PCI, percutaneous coronary intervention; PREDICTION, A Prospective, Multicenter, Randomized Trial of New Cutting Balloon Versus Conventional Balloon for the Treatment of Severe Calcified Lesion; PREPARE‐CALC, Comparison of Strategies to Prepare Severely Calcified Coronary Lesions; RCA, right coronary artery; ROLLER COASTR‐EPIC22, Rotational Atherectomy, Lithotripsy or Laser for the Treatment of Calcified Stenosis; ROTAXUS, Rotational Atherectomy Prior to Taxus Stent Treatment for Complex Native Coronary Artery Disease; SD, standard deviation; Short‐CUT, Shockwave Lithotripsy Compared to Cutting Balloon Treatment in Calcified Coronary Artery Disease; SONAR, ShOckwave ballooN or Atherectomy with Rotablation in calcified coronary artery lesions; VICTORY, Value of IVL Compared To OPN Non‐Compliant Balloons for Treatment of RefractorY Coronary Lesions.

In addition to the trial endpoints and characteristics (Tables S3 and S4) and baseline patient characteristics (Table 1), Table S6 summarizes the additional potential effect modifiers that were evaluated in order to support the assessment of the transitivity assumption.

### 3.1. Primary Outcome

**Figure 2:**
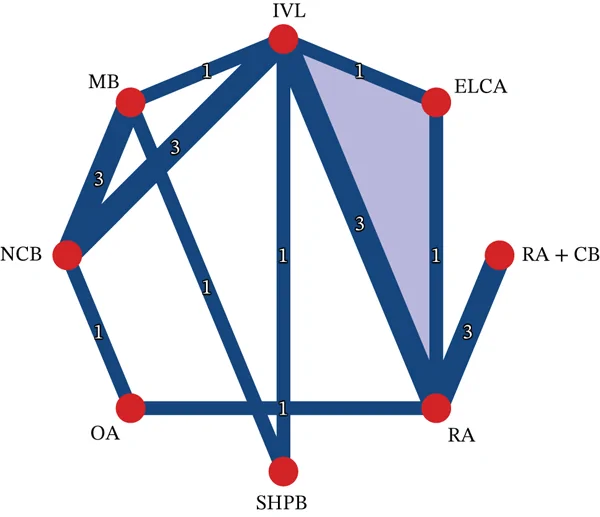
Network plot for minimal stent area by calcium‐modification strategies. The violet‐shaded area represents the devices directly connected through evidence from one three‐armed randomized trial. The modified balloon category for lesion preparation includes both cutting balloons and scoring balloons. CB, cutting balloon; ELCA, excimer laser coronary angioplasty; IVL, intravascular lithotripsy; MBs, modified balloons; NCBs, noncompliant balloons; OA, orbital atherectomy; SHPB, super‐high pressure balloon; RA, rotational atherectomy.

**Figure 3:**
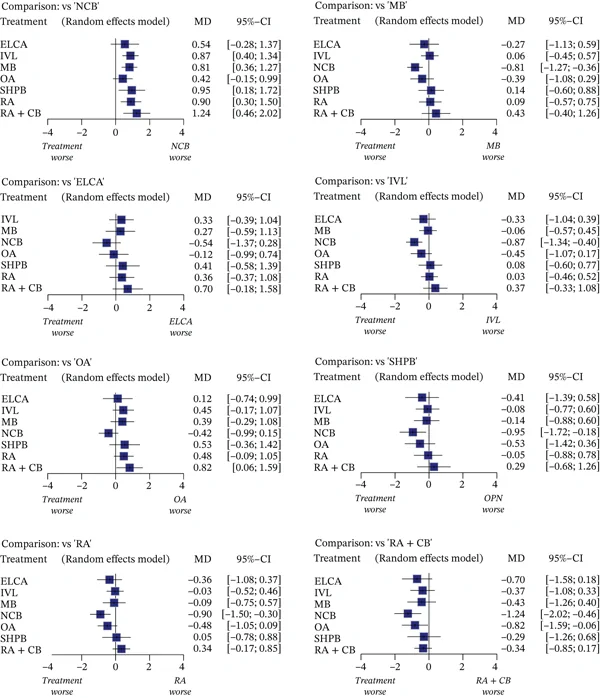
Mean differences for minimal stent area by calcium‐modification strategies. CI, confidence interval; CB, cutting balloon; ELCA, excimer laser coronary angioplasty; IVL, intravascular lithotripsy; MBs, modified balloons; MD, mean difference; NCBs, noncompliant balloons; OA, orbital atherectomy; SHPB, super high‐pressure balloons; RA, rotational atherectomy.

**Figure 4:**
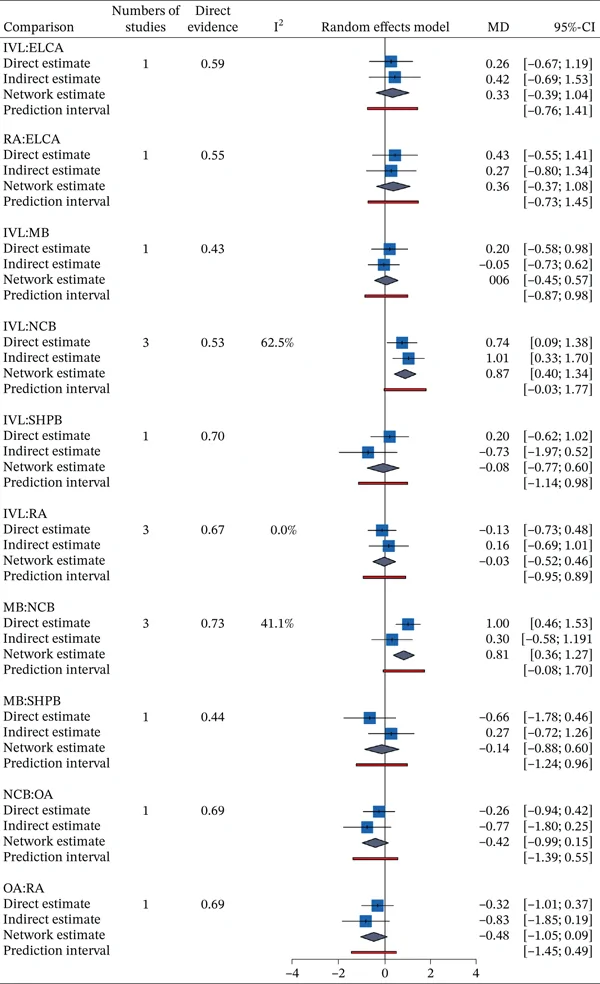
Node‐split model analysis for minimal stent area by calcium‐modification strategies. Node‐splitting analysis displaying, for each treatment comparison, the direct estimate (derived exclusively from head‐to‐head randomized trials), the indirect estimate (derived from the remaining evidence in the network through alternative comparison pathways), and the overall network estimate obtained under a random‐effects model. The “direct evidence” column represents the proportion of information contributing to the network estimate that originates from direct comparisons, rather than a clinical outcome measure. CI, confidence interval; ELCA, excimer laser coronary angioplasty; IVL, intravascular lithotripsy; MBs, modified balloons; MD, mean difference; NCBs, noncompliant balloons; OA, orbital atherectomy; SHPB, super high‐pressure balloon; RA, rotational atherectomy.

The network plot for the primary outcome is shown in Figure 2. All calcium‐modification strategies were directly compared with at least two others, except for RA plus CB. Overall, MSA was on average 6.08 mm^2^ and ranged from 4.45 to 8.15 mm^2^. When compared with NCB, MSA was significantly larger with all calcium‐modification strategies except ELCA (MD: 0.54 mm^2^; 95% CI: [−0.28, 1.37]; *p* = 0.198) and OA (0.42 mm^2^ [−0.15, 0.99]; *p* = 0.150, Figure 3). The MD of MSA values showed that IVL, SHPB, RA, and RA plus CB performed significantly better than other calcium‐modification strategies. This was further supported by rankogram analysis (Table 2). However, there was significant statistical heterogeneity for this outcome (*I* ^2^: 54.8%; *p* = 0.009). Notwithstanding this, the node‐splitting method did not reveal significant disagreement between direct and indirect evidence (Figure 4), and the presence of publication bias was ruled out both visually (Figure S3) and statistically (Egger′s test *p* = 0.192). Interestingly, the exclusion of trials with a high risk of bias in one or more components of the RoB 2 tool had little effect on the direction of the treatment effect for MSA. In fact, this remained consistent with the complete analysis, without significant heterogeneity (*I* ^2^: 6.5%; *p* = 0.378, Figure S4 and Table S7). The sensitivity analysis, which considered only studies with an IVI‐based definition of calcification, revealed that IVL and MB were the only calcium‐modification strategies to demonstrate a significant improvement in MSA compared to NCB. All the other calcium‐modification strategies were found to be comparable to NCB with regard to MSA (Figure S5 and Table S7). The sensitivity analysis excluding the Evaluation of Treatment Strategies for Severe Calcific Coronary Arteries: Orbital Atherectomy vs. Conventional Angioplasty Technique Prior to Implantation of Drug‐Eluting Stents (ECLIPSE) trial yielded results consistent with the primary analysis, with no meaningful changes in the direction or significance of the network estimates and net ranking (Figure S6).

**Table 2:** Net ranking for intravascular imaging and angiographic outcomes by calcium‐modification strategies.

Calcium‐modification strategy	*p* score for MSA^a^	*p* score for DS	Calcium‐modification strategy
RA + CB	0.1084	0.8915	RA + CB
SHPB	0.3249	0.8280	SHPB
RA	0.3602	0.5768	RA
IVL	0.3809	0.4878	IVL
MB	0.4391	0.3582	**ELCA**
ELCA	0.6569	0.3101	**MB**
OA	0.7561	0.2970	OA
NCB	0.9736	0.2508	NCB

*Note:* Devices whose ranking changed compared to the primary outcome are shown in bold.

Abbreviations: CB, cutting balloon; DS, diameter stenosis; ELCA, excimer laser coronary angioplasty; IVI, intravascular imaging; IVL, intravascular lithotripsy; MB, modified balloon; MI, myocardial infarction; MSA, minimal stent area; NCB, noncompliant balloon; OA, orbital atherectomy; RA, rotational atherectomy; SHPB, super high‐pressure balloon; ST, stent thrombosis.

^a^For MSA, a *p* score of 1 indicates the worst performance and 0 the best; conversely, for DS, a *p* score of 1 indicates the best performance and 0 the worst.

### 3.2. Secondary Angiographic Outcome

Post‐PCI DS was on average 9.56% and ranged from 4.30 to 31.76. Compared to NCB, only RA plus CB (−3.38% [−6.69, −0.07]; *p* = 0.045) barely led to significantly less DS after PCI. Consistent results were observed when it was compared with MB (RA plus CB, −2.86% [−5.63, −0.10]; *p* = 0.043; Figure S7). These findings were further supported by the rankogram analysis (Table 2).

### 3.3. Secondary Clinical Outcomes

Overall, all‐cause death occurred in 168 (3.96%) patients, while MI, ST, and repeat revascularization occurred in 318 (7.30%), 24 (0.62%), and 133 (3.42%) patients, respectively. None of the calcium‐modification strategies resulted in any significant differences with regard to all‐cause death, MI, ST, or repeat revascularization (Figure S8 and Table S8). These results remained consistent in the sensitivity analyses according to follow‐up duration, IVI utilization to define the degree of coronary calcification or to guide PCI. The analysis expressing the clinical outcomes as IRR showed no significant change in the direction of risk estimates (data not shown).

### 3.4. Secondary Procedural Outcomes

Coronary perforation was reported in 66 (1.43%) patients. No significant differences in the incidence of coronary perforations were observed across calcium‐modification strategies (Figure S9A and Table S8). Procedural success was achieved in 3765 (85.05%) patients. SHPB and RA‐based strategies demonstrated significantly more procedural success, especially compared to NCB (SHPB RR: 1.11; 95% CI: [1.02–1.21]; *p* = 0.014; RA: 1.13 [1.07–1.20]; *p* < 0.001; RA plus CB: 1.14 [1.04–1.25]; *p* = 0.006), MB (SHPB: 1.12 [1.04–1.22]; *p* = 0.005; RA: 1.14 [1.08–1.21]; *p* < 0.001; RA plus CB: 1.15 [1.05–1.26]; *p* = 0.003), and OA (SHPB: 1.12 [1.01–1.23]; *p* = 0.033; RA: 1.14 [1.05–1.23]; *p* = 0.002; RA plus CB: 1.14 [1.03–1.27]; *p* = 0.015; Figure S9B and Table S8).

## 4. Discussion

To the best of our knowledge, this is the first network meta‐analysis to compare IVI, procedural, and clinical outcomes of calcium‐modification strategies for treating calcified coronary lesions among all the randomized trials available. The main findings are as follows:

1. In terms of MSA after PCI, IVL, SHPB, and RA‐based techniques were among the highest performing calcium‐modification strategies compared with NCB. Additionally, SHPB and RA‐based techniques consistently achieved higher procedural success rates.
2. In contrast to the significant improvements observed in imaging, angiographic, and procedural outcomes, no calcium‐modification strategy resulted in a significant effect on clinical outcomes.

Firstly, unlike recent meta‐analyses dealing with a similar research topic [39, 40], this study incorporated the most recent randomized trials that were specifically designed to compare calcium‐modification strategies. In this regard, MSA was selected as the primary outcome of the present analysis. Although MSA is a surrogate outcome based on imaging findings, its prognostic value has been demonstrated in various scenarios, including in patients with calcified coronary lesions [41]. Notably, there is a well‐known inverse correlation between plaque volume and MSA [42]. While plaque volume tends to decrease following PCI, this effect depends on plaque composition [43]. For example, when soft plaques are present, stents can be expanded more effectively, as some of the plaque content may be compressed, thereby decreasing plaque volume. Conversely, this is not the case for calcified coronary lesions, which highlights the importance of appropriate calcium modification. The present analysis indirectly emphasizes the importance of reducing atheroma burden, suggesting that techniques capable of modifying vessel compliance at the calcium site could successfully improve stent expansion. In this regard, it is important to note that we found balloon‐based and RA‐based strategies outperform NCB. The results were confirmed in the analysis restricted to trials using IVI to define coronary calcification, contradicting recent evidence, in which NCB performed better than expected [14, 34].

Despite the clear advantages in terms of imaging, angiographic, and procedural outcomes, the lack of significant clinical differences between the evaluated devices may be due to a number of factors. It is worth noting that only three out of the 22 included trials had sufficient statistical power to detect differences in clinical outcomes [21, 34, 37]. Most trials assessing calcium‐modification strategies had a small sample size. This could have resulted in an underestimation of clinical events. Furthermore, the follow‐up duration was limited. In fact, only six studies reported 12‐month results [20, 26, 33–36], and only two extended the follow‐up out to 24 months [27, 28]. This could potentially have masked the long‐term clinical benefits of the evaluated strategies. In fact, the recent long‐term follow‐up of the Comparison of Strategies to Prepare Severely Calcified Coronary Lesions (PREPARE‐CALC) trial demonstrated a significant reduction in target lesion revascularization in the RA group after 5 years, as compared to MB [44]. This benefit was not apparent at 9 [30] or 24 months [45], highlighting the importance of extended follow‐up in this subset of patients.

Interestingly, when the analysis of the primary outcome was restricted to trials using an IVI‐based definition of coronary calcification, only IVL and MB were found to be superior to NCB in terms of MSA. This aspect merits careful consideration. The use of IVI to guide treatment in procedural planning and optimization for calcified lesions has been shown to enhance the modification of calcified coronary lesions, thereby reducing the risk of stent failure in different settings [46, 47]. In this context, IVL is a technology that disrupts both intimal and medial calcium and has demonstrated favorable safety and efficacy in severely calcified coronary lesions in previous aggregate analyses [48, 49]. Although similar to other balloon‐based strategies, its unique mechanism sets it apart. Interestingly, the present analysis showed that IVL and MB had comparable MSA, indicating similar effectiveness. Despite CB and SB being grouped together to reduce heterogeneity for the present analysis, their mechanisms remain distinct [50, 51]. Additionally, the use of CB with high‐pressure inflation has the potential to reach deeper calcium deposits. Although the underlying mechanisms differ, this ability to affect deeper calcium layers, also characteristic of IVL, may contribute to the similar results observed between the two approaches [18, 33]. However, the lack of individual patient data means we cannot determine whether the findings observed in this subgroup of patients were due to the smaller sample size or to the devices being better sized due to availability of baseline IVI, which could have contributed to better modification of calcified lesions and improved stent expansion at the end of the PCI procedure.

Ultimately, the combination of RA and CB appeared to be associated with higher procedural success than other calcium‐modification strategies, without an apparent increase in adverse events. However, while this observation is consistent with the current trend of combining calcium‐modification strategies for complex coronary lesions, it remains exploratory due to the small number of patients involved and should be confirmed in larger studies [52]. At the same time, it reflects the growing recognition that different devices, with their distinct mechanisms of action, may act synergistically to improve mechanical stent performance in patients with calcified coronary lesions. RA has historically been the most widely used of these strategies and has been shown to increase procedural success compared to standard strategies or MBs [28, 30]. Our results are consistent with previous studies using RA‐based techniques [28, 30, 32]. However, RA is not considered the preferred approach in several anatomical scenarios, particularly in the presence of high vessel tortuosity, bifurcated lesions, and small‐sized coronaries due to technical challenges and an increased procedural risk [53–55]. Furthermore, compared to balloon‐based strategies, RA has a longer learning curve and requires additional procedural steps.

### 4.1. Study Limitations

There are several limitations to this study that should be acknowledged. Although the assessment of transitivity did not identify clinically relevant imbalances in the main potential effect modifiers, the network was well connected, and node‐splitting analysis showed no significant inconsistency between direct and indirect evidence; transitivity remains an inherently untestable assumption of network meta‐analysis. Furthermore, the CINeMA assessment rated comparisons supported exclusively by indirect evidence as having low confidence. Because this analysis was based on aggregate trial‐level data, residual confounding due to unmeasured or incompletely reported effect modifiers cannot be excluded. Consequently, comparisons relying exclusively on indirect evidence should be interpreted with caution and considered hypothesis‐generating, as both the direction and the magnitude of any residual confounding cannot be predicted. In addition, the summary estimates for the primary outcome showed significant statistical heterogeneity. However, multiple sensitivity analyses substantially reduced the heterogeneity (e.g., by excluding studies at high risk of bias) rendering heterogeneity no longer significant while preserving the consistency and direction of the effect estimates. Several factors may have contributed to heterogeneity in this outcome. In particular, it is important to acknowledge that definitions of MSA were not completely uniform across studies, and measurements were obtained using different IVI modalities, including OCT and IVUS, which may yield slightly different absolute measurements despite their overall good agreement. However, the selection of postprocedural MSA as the primary endpoint was driven by the fact that only a limited number of randomized trials evaluating calcium‐modification strategies were adequately powered to detect differences in clinical outcomes. Therefore, MSA was selected as the most consistently reported surrogate endpoint across the available evidence. Another important limitation is that this is a trial‐level analysis, which has inherent constraints. Specifically, calcium‐modification strategies could have been used in combination for individual patients. Although the proportion of cases involving such combinations was low due to protocol restrictions, it should be recognized that adjustment for patient‐ or procedure‐level factors is not possible given the nature of this analysis. Similarly, the NCB group comprised a heterogeneous range of treatment strategies. Variations in balloon sizing, inflation pressures, operator technique, and adjunctive lesion preparation across trials may therefore have contributed to heterogeneity and partially influenced the observed outcomes. The composition of the available evidence also deserves consideration. The ECLIPSE trial alone accounted for approximately 40% of the overall study population and may therefore have exerted a substantial influence on the network estimates [34]. However, the sensitivity analysis excluding this trial yielded results consistent with the primary analysis, without meaningful changes in the network estimates. In addition, many of the trials investigating RA included balloon uncrossable lesions, which are clearly unsuitable for balloon‐based and other atheroablative strategies. This may have unpredictably biased the results. Furthermore, the definitions of clinical and procedural outcomes, particularly MI and procedural success, as well as of severe calcifications, varied substantially across the included studies, potentially contributing to clinical heterogeneity and affecting the comparability of outcome estimates. Next, data from 4 of the 22 included trials were extracted from conference abstracts. Although excluding gray literature can lead to exaggerated estimates of the treatment effect [56], unpublished studies often provide limited methodological detail and may change prior to full publication. However, excluding studies deemed to be at high risk of bias (including conference abstracts) produced consistent results, supporting the robustness of our findings. Finally, cutting and SBs were grouped together within the MB balloon category. Although these devices share similar structural characteristics, differences in their procedural use and operator‐dependent handling may have introduced heterogeneity into this treatment group [50]. In addition, although significant benefits were observed with SHPB and RA plus CB, whereas no efficacy emerged by using ELCA, the current body of evidence for these devices is limited to a small number of randomized trials, a fact that should be acknowledged when interpreting the results.

## 5. Conclusions

This study is the first to provide a comprehensive assessment of IVI, angiographic, procedural, and clinical outcomes across all calcium‐modification strategies for which randomized trials are available. Several strategies demonstrated superior mechanical performance in comparison to NCBs in treating calcified coronary lesions, particularly balloon‐based and RA‐based strategies. However, these advantages did not result in significant differences in clinical outcomes. Further randomized trials with adequate statistical power to assess clinical outcomes are needed to investigate the clinical performance of calcium‐modification strategies.

**Nomenclature**
ELCA: excimer laser coronary atherectomy
IVI: intravascular imaging
IVL: intravascular lithotripsy
MB: modified balloon
MD: mean difference
MSA: minimal stent area
NCB: noncompliant balloon
OA: orbital atherectomy
RA: rotational atherectomy
SHPB: super high‐pressure balloon

## Funding

No funding was received for this manuscript. Open Access funding enabled and organized by Projekt DEAL.

## Disclosure

This systematic review and network meta‐analysis was performed and reported according to the PRISMA guidelines.

## Ethics Statement

This study was conducted in accordance with the ethical principles outlined in the Declaration of Helsinki (2013). Given its retrospective, trial‐level nature based on anonymized published data, no institutional ethical approval or informed consent was required.

## Conflicts of Interest

S.C. reports consulting/lecture fees from SIS Medical AG and Translumina GmbH; lecture/advisory board fees from Abbott Vascular; lecture fees from B.Braun, Shockwave Inc., SIS Medical AG, Teleflex and Translumina GmbH; financial support for attending meetings or travel expenses from Abbott Vascular, SIS Medical AG, and Translumina GmbH; and an educational grant to the institution of employment from Abbott Vascular and SIS Medical AG, not related to the current work. F.S. reports lecture fees from Translumina GmbH, not related to the current work. F.V. reports lecture fees from Abbott Vascular, AstraZeneca, and Translumina GmbH, not related to the current work. N.T.O. reports research grants through his institution from Abbott and Shockwave. The remaining authors have no conflicts of interest to declare.

## Supporting information


**Supporting Information** Additional supporting information can be found online in the Supporting Information section. Table S1 PRISMA 2020 Checklist. Table S2. Full electronic database search process. Table S3. Definitions of main outcomes according to the original protocols of the included trials. Table S4. Main trial characteristics. Table S5. Summary of the CINeMA framework domains. Table S6. Additional trial characteristics for transitivity evaluation. Table S7. Sensitivity analysis‐based rankogram (*p* score) for the primary outcome. Table S8. Rankogram analyses (*p* score) for secondary clinical and procedural outcomes. Figure S1. PRISMA 2020 flow chart for study selection. Figure S2. Risk‐of‐Bias assessment with Cochrane RoB tool v2 for randomized trials. Figure S3. Funnel plot distribution of mean differences in minimal stent area for each calcium‐modification device comparison. Figure S4. Sensitivity analysis of the primary outcome excluding high risk of bias studies. Figure S5. Sensitivity analysis of the primary outcome including only studies using an IVI‐based definition of severe calcification. Figure S6. Sensitivity analysis of the primary outcome excluding the ECLIPSE trial. Figure S7. Post‐PCI diameter stenosis network analysis. Figure S8. League tables for secondary clinical outcomes. Figure S9. League tables for secondary procedural outcomes.

## Data Availability

The data supporting the findings of this study are available from the corresponding author upon reasonable request.
