# Supplementary material for: Comparative Performance of Calcium‐Modification Strategies for Coronary Lesions: A Network Meta‐Analysis of Randomized Trials
Source: Cardiovasc Ther. 2026 Aug 30;2026:9434123. doi: 10.1155/cdr/9434123 (PMC13527234; doi:10.1155/cdr/9434123)
Supplement: Supplementary file 1 [file CDR-2026-9434123-s001.docx]

**Supplementary Material**

**Comparative performance of calcium-modification strategies for coronary lesions: a network meta-analysis of randomized trials**

[Supplemental Tables	2](#_Toc235353846)

[Table S1. PRISMA 2020 Checklist	2](#_Toc235353847)

[Table S2. Full electronic database search process	6](#_Toc235353848)

[Table S3. Definitions of main outcomes according to the original protocols of the included trials	7](#_Toc235353849)

[Table S4. Main trial characteristics	15](#_Toc235353850)

[Table S5. Summary of the CINeMA framework domains	29](#_Toc235353851)

[Table S6. Additional trials characteristics for transitivity evaluation	31](#_Toc235353852)

[Table S7. Sensitivity Analysis-Based Rankogram (P-Score) for the Primary Outcome	32](#_Toc235353853)

[Table S8. Rankogram analyses (P-Score) for secondary clinical and procedural outcomes	33](#_Toc235353854)

[Supplemental Figures	34](#_Toc235353855)

[Figure S1. PRISMA 2020 flow chart for study selection	34](#_Toc235353856)

[Figure S2. Risk-of-Bias Assessment with Cochrane RoB tool v2 for randomized trials	35](#_Toc235353857)

[Figure S3. Funnel plot distribution of mean differences in minimal stent area for each calcium modification device comparison	36](#_Toc235353858)

[Figure S4. Sensitivity analysis of the primary outcome excluding High Risk of Bias Studies	37](#_Toc235353859)

[Figure S5. Sensitivity analysis of the primary outcome including only studies using an IVI-based definition of severe calcification	38](#_Toc235353860)

[Figure S6. Sensitivity analysis of the primary outcome excluding the ECLIPSE trial	39](#_Toc235353861)

[Figure S7. Post-PCI diameter stenosis Network analysis	40](#_Toc235353862)

[Figure S8. League tables for secondary clinical outcomes	41](#_Toc235353863)

[Figure S9. League tables for secondary procedural outcomes	42](#_Toc235353864)

# Supplemental Tables

## Table S1. PRISMA 2020 Checklist

| **Section and Topic** | **Item #** | **Checklist item** | **Page where item is reported** |
| --- | --- | --- | --- |
| **TITLE** | | |  |
| Title | 1 | Identify the report as a systematic review. | 1 |
| **ABSTRACT** | | |  |
| Abstract | 2 | See the PRISMA 2020 for Abstracts checklist. | 2 |
| **INTRODUCTION** | | |  |
| Rationale | 3 | Describe the rationale for the review in the context of existing knowledge. | 4 |
| Objectives | 4 | Provide an explicit statement of the objective(s) or question(s) the review addresses. | 4 |
| **METHODS** | | |  |
| Eligibility criteria | 5 | Specify the inclusion and exclusion criteria for the review and how studies were grouped for the syntheses. | 5 |
| Information sources | 6 | Specify all databases, registers, websites, organisations, reference lists and other sources searched or consulted to identify studies. Specify the date when each source was last searched or consulted. | 5 |
| Search strategy | 7 | Present the full search strategies for all databases, registers and websites, including any filters and limits used. | 6 (Supp.) |
| Selection process | 8 | Specify the methods used to decide whether a study met the inclusion criteria of the review, including how many reviewers screened each record and each report retrieved, whether they worked independently, and if applicable, details of automation tools used in the process. | 5 |
| Data collection process | 9 | Specify the methods used to collect data from reports, including how many reviewers collected data from each report, whether they worked independently, any processes for obtaining or confirming data from study investigators, and if applicable, details of automation tools used in the process. | 5 |
| Data items | 10a | List and define all outcomes for which data were sought. Specify whether all results that were compatible with each outcome domain in each study were sought (e.g. for all measures, time points, analyses), and if not, the methods used to decide which results to collect. | 6 |
|  | 10b | List and define all other variables for which data were sought (e.g. participant and intervention characteristics, funding sources). Describe any assumptions made about any missing or unclear information. | 5-6 |
| Study risk of bias assessment | 11 | Specify the methods used to assess risk of bias in the included studies, including details of the tool(s) used, how many reviewers assessed each study and whether they worked independently, and if applicable, details of automation tools used in the process. | 6 |
| Effect measures | 12 | Specify for each outcome the effect measure(s) (e.g. risk ratio, mean difference) used in the synthesis or presentation of results. | 6-7 |
| Synthesis methods | 13a | Describe the processes used to decide which studies were eligible for each synthesis (e.g. tabulating the study intervention characteristics and comparing against the planned groups for each synthesis (item #5)). | 5 |
|  | 13b | Describe any methods required to prepare the data for presentation or synthesis, such as handling of missing summary statistics, or data conversions. | 6 |
|  | 13c | Describe any methods used to tabulate or visually display results of individual studies and syntheses. | 5-6 |
|  | 13d | Describe any methods used to synthesize results and provide a rationale for the choice(s). If meta-analysis was performed, describe the model(s), method(s) to identify the presence and extent of statistical heterogeneity, and software package(s) used. | 6 |
|  | 13e | Describe any methods used to explore possible causes of heterogeneity among study results (e.g. subgroup analysis, meta-regression). | 6-7 |
|  | 13f | Describe any sensitivity analyses conducted to assess robustness of the synthesized results. | 6-7 |
| Reporting bias assessment | 14 | Describe any methods used to assess risk of bias due to missing results in a synthesis (arising from reporting biases). | 6-7 |
| Certainty assessment | 15 | Describe any methods used to assess certainty (or confidence) in the body of evidence for an outcome. | 6-7 |
| **RESULTS** | | |  |
| Study selection | 16a | Describe the results of the search and selection process, from the number of records identified in the search to the number of studies included in the review, ideally using a flow diagram. | 31 (Supp.) |
|  | 16b | Cite studies that might appear to meet the inclusion criteria, but which were excluded, and explain why they were excluded. | 7 |
| Study characteristics | 17 | Cite each included study and present its characteristics. | 7-8 |
| Risk of bias in studies | 18 | Present assessments of risk of bias for each included study. | 7 |
| Results of individual studies | 19 | For all outcomes, present, for each study: (a) summary statistics for each group (where appropriate) and (b) an effect estimate and its precision (e.g. confidence/credible interval), ideally using structured tables or plots. | Table 1 |
| Results of syntheses | 20a | For each synthesis, briefly summarise the characteristics and risk of bias among contributing studies. | 32 (Supp.) |
|  | 20b | Present results of all statistical syntheses conducted. If meta-analysis was done, present for each the summary estimate and its precision (e.g. confidence/credible interval) and measures of statistical heterogeneity. If comparing groups, describe the direction of the effect. | 8-9 |
|  | 20c | Present results of all investigations of possible causes of heterogeneity among study results. | 7 |
|  | 20d | Present results of all sensitivity analyses conducted to assess the robustness of the synthesized results. | 7-9 |
| Reporting biases | 21 | Present assessments of risk of bias due to missing results (arising from reporting biases) for each synthesis assessed. | 33 (Supp.) |
| Certainty of evidence | 22 | Present assessments of certainty (or confidence) in the body of evidence for each outcome assessed. | 7-9 |
| **DISCUSSION** | | |  |
| Discussion | 23a | Provide a general interpretation of the results in the context of other evidence. | 10-12 |
|  | 23b | Discuss any limitations of the evidence included in the review. | 12-13 |
|  | 23c | Discuss any limitations of the review processes used. | 13 |
|  | 23d | Discuss implications of the results for practice, policy, and future research. | 11-12 |
| **OTHER INFORMATION** | | |  |
| Registration and protocol | 24a | Provide registration information for the review, including register name and registration number, or state that the review was not registered. | 5 |
|  | 24b | Indicate where the review protocol can be accessed, or state that a protocol was not prepared. | 5 |
|  | 24c | Describe and explain any amendments to information provided at registration or in the protocol. | 5 |
| Support | 25 | Describe sources of financial or non-financial support for the review, and the role of the funders or sponsors in the review. | 14 |
| Competing interests | 26 | Declare any competing interests of review authors. | 14 |
| Availability of data, code and other materials | 27 | Report which of the following are publicly available and where they can be found: template data collection forms; data extracted from included studies; data used for all analyses; analytic code; any other materials used in the review. | 14 |

## Table S2. Full electronic database search process

| EMBASE AND MEDLINE | | |
| --- | --- | --- |
| Search line | **Search Terms** | **No. citations** |
| 1 | **'percutaneous coronary intervention'**:ab,ti AND ([embase]/lim OR [medline]/lim) | 75287 |
| 2 | **'coronary artery disease'**:ab,ti AND ([embase]/lim OR [medline]/lim) | 172537 |
| 3 | **'coronary angiography'**:ab,ti AND ([embase]/lim OR [medline]/lim) | 68834 |
| 4 | **'transluminal coronary angioplasty'**:ab,ti AND ([embase]/lim OR [medline]/lim) | 8196 |
| 5 | **'drug eluting stent'**:ab,ti AND ([embase]/lim OR [medline]/lim) | 11592 |
| 6 | **'cardiovascular disease'**:ab,ti AND ([embase]/lim OR [medline]/lim) | 264332 |
| 7 | **'coronary artery calcification'**:ab,ti AND ([embase]/lim OR [medline]/lim) | 5655 |
| 8 | **'intravascular lithotripsy'**:ab,ti AND ([embase]/lim OR [medline]/lim) | 944 |
| 9 | **'atherectomy device'**:ab,ti AND ([embase]/lim OR [medline]/lim) | 278 |
| 10 | **'atherectomy catheter'**:ab,ti AND ([embase]/lim OR [medline]/lim) | 142 |
| 11 | **'cutting balloon catheter'**:ab,ti AND ([embase]/lim OR [medline]/lim) | 29 |
| 12 | **'scoring balloon'**:ab,ti AND ([embase]/lim OR [medline]/lim) | 290 |
| 13 | **'rotational atherectomy'**:ab,ti AND ([embase]/lim OR [medline]/lim) | 2294 |
| 14 | **'orbital atherectomy'**:ab,ti AND ([embase]/lim OR [medline]/lim) | 560 |
| 15 | #1 AND #7 | 226 |
| 16 | #8 OR #9 OR #10 OR #11 OR #12 OR #13 OR #14 | 4016 |
| 18 | #7 OR #16 | **9500** |
| Conference abstracts and presentations were identified by manually screening the online programs of major interventional cardiology meetings (including TCT, EuroPCR, and ESC Congress) from the most recent year within the predefined search period. These records were not retrieved through database indexing but through a targeted manual search. | | |

## Table S3. Definitions of main outcomes according to the original protocols of the included trials

| Trial | MSA | Death | MI | ST | Repeat revascularization | Procedural success |
| --- | --- | --- | --- | --- | --- | --- |
| BALI | The smallest stent area measured in the entire stented segment | Cardiac death | MI: 4^th^ UDMI  Peri-procedural MI: ARC-2 | ARC-2 | Clinically-driven revascularization | Successful stent delivery and OCT assessed residual area stenosis ≥20% |
| BASIL | N/R | Death of any cause | 4^th^ UDMI | N/R | N/R | Successful stenting without the use of bailout calcium-modification strategies, without major angiographic complications, and with angiographic evidence of residual DS <20% |
| COPS | Smallest cross‐sectional area of the DES in correspondence to the calcified lesion | N/R | N/R | N/R | ARC-2 | Successful device delivery, inflation with adequate lesion preparation, and the absence of vessel rupture/perforation |
| DIRO | N/R | ARC-2 | ARC-2 | ARC-2 | ARC-2 | N/R |
| ECLIPSE | N/R | Death of any cause | 3^rd^ UDMI and SCAI for periprocedural MI | ARC (modified) | Ischemia-driven TVR (inclusive of the target lesion) after completion of the procedure | Successful stent delivery with final Core Lab defined TIMI 3 flow and angiographic in-stent DS ≤20%, and without any of: stent loss, coronary perforation, or intra-procedural death |
| Elyamani et al. | N/R | Death of any cause | Based on the detection of hs-cTn levels above the 99th percentile of URL value for a healthy reference population, together with symptoms and/or ischemic ECG changes | ARC-2 | Any repeat PCI of the target lesion or bypass surgery of the target vessel | Post-procedural stenosis≤20% and TIMI flow grade III |
| EXIT-CALC | N/R | Cardiac death | N/R | ARC-2 | N/R | N/R |
| Han et al. | N/R | N/R | N/R | N/R | N/R | N/R |
| ICARE | In-stent minimal cross-sectional area as assessed by OCT | Death of any cause | MI related to target vessel including preriprocedural MI according to ARC 2 | Definite according to ARC-2 | Any revascularization | N/R |
| ISAR-CALC | N/R | Cardiac death | Target vessel MI | N/R | N/R | Angiographic success without the occurrence of MACE up to 30 days |
| Li et al. | N/R | Death of any cause | 3^rd^ UDMI | N/R | Revascularization during the follow-up period due to restenosis within the target lesion | Periprocedural rates of successful stent implantation and periprocedural complications (including coronary dissection, coronary perforation, coronary slow flow or no-reflow, ST, and stent dislodgment), balloon dilation, and PCI |
| PREDICTION^*^ | The Smallest stent area measured at the site of maximum calcification by IVUS | N/R | N/R | N/R | N/R | N/R |
| PREPARE CALC | The average of 2 orthogonal views (when possible) of the narrowest point within the area of assessment, here within the stent boundaries | Death of any cause | ARC-2 | ARC-2 | Any repeat PCI of the target lesion or bypass surgery of the target vessel | Successful stent delivery and expansion with attainment of <20% in-stent residual stenosis of the target lesion in the presence of TIMI 3 flow without crossover or stent failure |
| ROLLER COASTR | N/R | Death of any cause | N/R | N/R | N/R | Angiographic success was defined as final TIMI flow grade 3 with residual stenosis <20% measured on QCA (also calculated with residual stenosis <30%). Procedural success was defined as angiographic success without severe procedural complications (death, perforation, flow-limiting dissection, acute vessel closure, or ST) |
| ROTA.Shock | N/R | N/R | N/R | N/R | N/R | N/R |
| ROTA-CUT | N/R | Death of any cause | Defined by the SCAI definition and 4^th^ UDMI including periprocedural myocardial infarction | Definite/probable (ARC-2) | N/R | N/R |
| ROTAXUS | N/R | Death of any cause | CK >3x the ULN with elevated CK-MB (any amount above the institution’s ULN) without new pathological Q waves (*non–Q-wave MI)*, or as chest pain or other acute symptoms consistent with myocardial ischemia and/or new pathological Q waves in ≥2 contiguous ECG leads in the absence of timely cardiac enzyme data and/or new pathological Q waves in ≥2 contiguous leads and elevation of cardiac enzymes (*Q-wave MI*) | ARC | Any reintervention inside the stent implanted during the index procedure or within 5 mm proximal or distal to the stent | *Angiographic success*: successful stent delivery and expansion with attainment of <20% in-stent residual stenosis of the target lesion in the presence of TIMI flow grade 3;  S*trategy success*: angiographic success without crossover or stent loss |
| Short-CUT^†^ | Post-procedural stent area at the point of maximum calcification as measured by intravascular imaging | Death of any cause | Periprocedural MI according to ARC definition | N/R | N/R | Stent delivery with a residual stenosis < 20% in the absence of significant angiographic complication with final TIMI 3 flow in the target vessel and the absence of intra-procedural death |
| SONAR^†^ | N/R | Death of any cause | 4^th^ UDMI (peri-procedural) | N/R | Any repeat revascularization of the target lesion | Angiographic success with/without in-hospital MACE |
| Tang et al. | N/R | Death of any cause | 3^rd^ UDMI | N/R | Repeat revascularization in the follow-up period due to restenosis, either within the target lesion or within the same coronary artery | N/R |
| VICTORY^†^ | N/R | Death of any cause | 4^th^ UDMI | ARC | Any repeat percutaneous or surgical intervention due to a stenosis or occlusion within the stent or within the 5 mm border proximal or distal to the stent | Angiographic success without any MACE (defined as cardiovascular death, target vessel-related MI, TIA/ stroke and/or repeat revascularization) up to 30 days after index PCI |
| Zou et al. | N/R | N/R | N/R | N/R | N/R | At the end of the procedure, angiographically confirmed residual stent stenosis ≤ 30%, TIMI 3 flow, and no occurrence of MACE (defined as cardiac death, target-vessel MI, TLR, cerebrovascular accident, ST) during hospitalization |
| *^*^Data previously presented at the EuroPCR 2025 Conference. ^†^Data previously presented at the TCT 2025 Conference. ARC: Academic Research Consortium, CV: Cardiovascular; DES: Drug-Eluting Stent, DS: Diameter Stenosis, HF: Heart Failure, MACE: Major Adverse Cardiovascular Events, MI: Myocardial Infarction, MSA: Minimum Stent Area, OCT: Optical Coherence Tomography, PCI: Percutaneous Coronary Intervention, QCA: Quantitative Coronary Angiography, SCAI: Society for Cardiovascular Angiography and Interventions, ST: Stent Thrombosis, STEMI: ST Elevated Myocardial Infarction; TIA: Transitory Ischemic Attack; TIMI: Thrombolysis In Myocardial Infarction, TLR: Target Lesion Revascularization, TVR: Target Vessel Revascularization, UDMI: Universal Definition of Myocardial Infarction, URL: Upper Reference Limit. Trial acronyms: BALI: Balloon Lithoplasty for Preparation of Severely Calcified Coronary Lesions, BASIL: Balloon Angioplasty versus Shockwave Intravascular Lithotripsy, COPS: Cutting Balloon to Optimize Predilatation for Stent Implantation, DIRO: direct comparison of RA vs OA for calcified lesions guided by OCT, ECLIPSE: Evaluation of Treatment Strategies for Severe CaLcIfic Coronary Arteries: Orbital Atherectomy vs. Conventional Angioplasty Technique Prior to Implantation of Drug-Eluting StEnts, EXIT-CALC: EXpansion of stents after Intravascular lithotripsy versus conventional predilatation in CALCified coronary arteries, ICARE: Intravascular Lithotripsy in Comparison to Rotational Atherectomy: An Evaluation by OFDI, ISAR-CALC: ComparIson of Strategies to PrepAre SeveRely CALCified Coronary Lesions, PREDICTION: A Prospective, Multicenter, Randomized Trial of New Cutting Balloon Versus Conventional Balloon for the Treatment of Severe Calcified Lesion, PREPARE-CALC: Comparison of Strategies to Prepare Severely Calcified Coronary Lesions, ROLLER COASTR-EPIC22: Rotational Atherectomy, Lithotripsy or Laser for the Treatment of Calcified Stenosis, ROTAXUS: Rotational Atherectomy Prior to Taxus Stent Treatment for Complex Native Coronary Artery Disease, Short-CUT: Shockwave Lithotripsy Compared to Cutting Balloon Treatment in Calcified Coronary Artery Disease, SONAR: ShOckwave ballooN or Atherectomy with Rotablation in calcified coronary artery lesions, VICTORY: Value of IVL Compared To OPN Non-Compliant Balloons for Treatment of RefractorY Coronary Lesions.* | | | | | | |

## Table S4. Main trial characteristics

| Trial | Design | Definition of severe calcification | Primary Outcome | Main inclusion criteria | Main exclusion criteria |
| --- | --- | --- | --- | --- | --- |
| BALI | Multicenter, open-label; IVL versus standard (1:1) | *Angiography*: radiopacities of the vessel wall visible on cine images before contrast injection on both sides of the vessel lumen in more than one projection; *OCT*: maximum calcium 1) angle >180° AND 2) thickness 0.5mm, AND 3) longitudinal length >5mm; *IVUS* (before lesion preparation): Maximum calcium angle >270° | Strategy failure (failed stent delivery, residual area stenosis ≥20% at OCT) after PCI, or target vessel failure | Age between 18 and <90 years; stable CAD or NSTE-ACS; PCI planned in severely calcified, non-occluded, de-novo lesion in native vessel; functional evidence of ischemia in the target vessel territory or stenosis ≥ 90% by visual estimate; RVD between 2.5 and 4 mm; ability to tolerate DAPT | Chronic total occlusion; severely calcified bifurcated lesion with expected need to use two stent technique; STEMI; planned later revascularization in non-study lesions or planned cardiovascular intervention ≤30 days after study intervention; LVEF <35%; eGFR <30 mL/min |
| BASIL | Single-center, open-label; IVL versus NCB (1:1) | *IVUS*: calcium arc ≥270°; *Angiography*: radiopacities without cardiac motion before contrast injection generally compromising both sides of the arterial lumen if the IVUS catheter was unable to cross the lesion for baseline assessment | Procedural success; freedom from in-hospital MACE | PCI with chronic or acute coronary syndromes and severely calcified coronary artery lesions  Age>18 years; stable CAD or NSTE-ACS; PCI planned in severely calcified, non-occluded, de-novo lesion in native vessel; RVD ≥2.5 mm | STEMI; cardiogenic shock; contraindication to DAPT for ≥6 months |
| COPS | Multicenter open‐label; CB versus NCB (1:1) | *IVUS:* More than 100° of calcium demonstrated at baseline IVUS | MSA at the level of the calcified segment (the smallest cross‐sectional area of the DES in correspondence to the calcified lesion) | Age >18 years old; severe de novo‐calcified lesions (baseline IVUS); RVD between 2.5 and 4.0 mm; suitable for PCI | Significant comorbidities precluding clinical follow‐up; contra‐indications to DAPT; platelet count < 100,000 × 109/L; planned major surgery within 1 year; Treatment of ISR; graft stenosis, thrombotic lesions |
| DIRO | Single-center; RA versus OA (1:1) | *OCT:* More than 180°  *Angiography:* moderate/severe | Stent expansion (minimum stent area divided by distal reference lumen area) | Age > 18 years old; stable coronary artery disease had to have a clinical indication for PCI; De novo calcified lesions | Ostial left main artery or ostial RCA lesions; childbearing potential; life expectancy of fewer than 12 months |
| ECLIPSE | Multicenter, open-label; OA versus conventional (1:1) stratified by intended treatment of single vs. multiple lesions and enrolling site | *Angiography:* presence of radiopacities noted without cardiac motion involving both sides of the arterial wall, with total calcium length ≥15 mm extending into the target lesion  *IVI*: ≥270 degrees of calcium in at least one imaging cross-section | 1) The acute post-procedural MSA at the site of maximal calcification assessed by OCT in a pre-specified cohort of approximately 500 patients; and 2) TVF: the composite of cardiac death, target vessel-related MI, or ischemia-driven TVR | **General:** Age > 18 years of age; a. stable ischemic heart disease or b. acute coronary syndrome (Non-STEMI or unstable angina), or c. stabilized recent STEMI (>48 hours prior to randomization procedure); **Angiographic:** 1. The target lesion(s) is a de novo (non-restenotic) coronary lesion; 2. a. stenosis ≥70% and <100%, or b. stenosis ≥50% and <70% with evidence of ischemia; 3. RVD ≥2.5mm and ≤4.0mm at the lesion site; 4. TIMI flow 2 or 3 at baseline; 5. evidence of severe calcium at the lesion site | **General:** 1. history of any cognitive or mental health status that would interfere with trial participation; 2. participating in or has plans to participate in any other investigational drug or device trial; 3. pregnancy; 4. receiving or scheduled to receive chemotherapy; 5. life expectancy of ≤ 12 months; 6. any prior PCI in the target vessel or its branches; 7. undergone a PCI procedure that is unsuccessful or with complications within 30 days prior to randomization; 8. Any cardiac intervention or cardiac surgery planned within 12 months; 9. major valve disease and underwent intervention within 30 days; 10. heart transplant; 11. Evidence of heart failure 12. known sensitivity to contrast media, 13. relative or absolute contraindication to DAPT; 14. history of a stroke or TIA within 6 months 15. history of bleeding diathesis; 16. active infection;  **Angiographic:** 1. Each target lesion requires treatment within or through an arterial or venous bypass graft; 2. angiographically visible or suspected thrombus; 3. severe or excessive tortuosity; 4. unprotected left main lesion, an aorto-ostial lesion 5. true bifurcation with a heavily calcified side branch, or if the side branch requires planned stenting or other intervention; 6. Angiographic or imaging evidence of a dissection in the target vessel(s) prior to randomization. 7. All 3 major epicardial coronary arteries require treatment, unless the treatment of 1 or 2 vessels is staged |
| Elyamani et al | Single center; RA versus NCB **(**1:1) | *Angiography:* Radiopacities noted without cardiac motion before contrast injection generally compromising both sides of the arterial lumen | Angiographic success (post-procedural stenosis≤20% and TIMI flow grade III) | Age >18 years old;  angiography proved coronary artery disease, having anginal symptoms and/or reproducible ischemia in the target area; angiography revealed de-novo lesion in a native coronary artery, RVD between 2.25 and 4.0 mm; luminal diameter reduction of 50-100% and had severe calcification | Myocardial infarction within 1-week; decompensated heart failure; limited long-term prognosis; target lesion in a coronary artery bypass graft or ISR; target vessel thrombus |
| EXIT-CALC | Single-center; IVL versus conventional and/or speciality balloons (1:1) | *OCT*: Calcium score of 4, defined as a maximum calcium angle of >180◦, maximum calcium thickness > 0.5 mm, and a minimal calcium length of 5 mm, as measured at the target lesion | Minimal stent expansion (smallest stent area divided by the average of proximal and distal stent area x100) | PCI of a calcified lesion in a native coronary artery; OCT: derived calcium score of 4; RVD between 2.5 and 4.0 mm | Severe congestive heart failure (NYHA 4); STEMI as indication for PCI; glomerular filtration rate < 30 ml/min/1.72m2; ISR, side branch >2.0 mm originating from the target lesion; contraindication for DAPT; life expectancy less than two years |
| Han e al | Single-center, RA + CB versus SB  (1:1) | *IVUS*: Strong echogenic clusters with acoustic shadows along the vessel wall with a max calc arc >270° | Intra-procedural complication, MACE. | Unstable angina (Chinese guidelines); initial assessment by Coronary CT; IVUS with calcific arch >270° Severe, ischemia-associated stenosis (e.g. >70%); combination of diabetes and hypertension under treatment; signed informed consent | Ulcerative, thrombotic, or occluded target lesion; severe tortuosity (>60°); target lesion in SVG; lesion with dissection before treatment; uncrossable lesion; LVEF <30%; combination of malignancy, hematological disorders, autoimmune disease, PAD, severe organ insufficiency; contraindication to PCI |
| ICARE | Multicenter, open-label; IVL versus RA (1:1) | *Angiography*: Evidence of calcification at the lesion with a B or C Mintz classification site. | Acute MSA (in-stent minimal cross-sectional area as assessed by OCT) | Age ≥ 18 years old; native coronary artery disease; single de novo target lesion stenosis of protected left main, or LAD, RCA, or LCX (or of their branches) with:  Stenosis of ≥70% and <100% or Stenosis ≥50% and <70% with evidence of ischemia; RVD ≥2.5 mm and ≤4.0 mm; lesion length ≤ 40 mm; TIMI flow 3 at baseline; evidence of calcification at the lesion with a B or C Mintz classification site; Ability to cross target lesion with a balloon | Age < 18 years; subject pregnant or nursing; refusing to participate in the study or unable to give informed consent; any comorbidity or condition which may reduce compliance with this protocol; participating in another research study involving an investigational agent (pharmaceutical, biologic, or medical device); unable to tolerate DAPT; allergy to imaging contrast media; experienced an acute MI (STEMI or non-STEMI) within 30 days prior to index procedure; NYHA class III or IV heart failure; history of a stroke or TIA; active peptic ulcer or upper gastrointestinal bleeding; active systemic infection; life expectancy of less than 1 year; planned non-coronary interventional or surgical structural heart procedures (e.g., TAVR, MitraClip, LAA or PFO occlusion...); high SYNTAX Score (≥33); definite or possible thrombus; evidence of aneurysm in target vessel within 10 mm; Unprotected left main; angiographic evidence of dissection |
| ISAR-CALC | Multicenter, assessor-blind, open-label; SHPB versus scoring balloon (1:1) | *Angiography*: according to the angiographic classification of Mintz et al | Expansion index ( minimum stent area divided by mean reference lumen area assessed with OCT) | Age ≥18 years; persistent angina despite optimal medical therapy and/or evidence of inducible ischaemia; proven coronary artery disease; de novo lesion in a native coronary artery; RVD between 2.25 and 4.00 mm; severe calcification of the target lesion; unsuccessful lesion preparation with standard non-compliant balloon | Myocardial infarction (within 1 week); target lesion located in a coronary artery bypass graft or ISR or aorto-ostial; target vessel thrombus; limited long-term prognosis |
| Li et al | Multicenter, open-label; RA + CB versus RA (1:1) | *IVUS*: calcium arc of ≥180° | Acute lumen gain. RA-associated periprocedural rates of successful stent implantation and periprocedural complications (including coronary dissection, coronary perforation, coronary slow flow or no-reflow, stent thrombosis, and stent dislodgment), balloon dilation, and PCI | At least one severe coronary stenosis ≥70 %, with at least one severely calcified lesion | Acute thrombosis with unstable hemodynamic status; ISR; extremely tortuous or angulated lesions or lesions with dissection before balloon expansion; SVG; other complications or comorbidities which were contraindicated for angiography or PCI |
| PREDICTION^*^ | Multicenter; CB versus conventional balloons (1:1) | *Angiography*: severely calcified de-novo coronary lesions | Final MSA at the maximum calcium segment | ≥ 20 years old; ischemic heart disease scheduled to receive coronary artery intervention for heavily calcified lesion | Lesion in the left main; STEMI (within 24 hours from onset); lesion in bypass graft; ISR; Chronic total occlusion lesion; cardiogenic shock; acute-phase cardiac failure; sustained ventricular arrhythmia; Ejection Fraction < 30%; severe valvular disease; or eGFR < 30mL/min/1.72m2, under non-dialysis; pregnant or lactating; history of hypersensitivity to contrast medium; life expectancy of less than one year |
| PREPARE CALC | Multicenter; MB versus RA (1:1) | *Angiography*: radiopacities noted without cardiac motion before contrast injection generally compromising both sides of the arterial lumen | Strategy success (successful stent delivery and expansion with attainment of <20% in-stent residual stenosis of the target lesion in the presence of TIMI 3 flow without crossover or stent failure) and in-stent LLL (difference between the postprocedure and 9-month in-stent angiographic minimal lumen diameter) | **Clinical:** Age > 18 years; proven coronary artery disease; anginal symptoms and/or reproducible ischemia; informed written consent; **Angiographic:** de-novo lesion in a native coronary artery; RVD between 2.25 and 4.0 mm; luminal diameter reduction of 50-100%; severe calcification of the target lesion | **Clinical:** Myocardial infarction within 1 week; decompensated heart failure; limited long term prognosis due to other conditions; **angiographic:** coronary artery bypass graft; ISR; target vessel thrombus |
| ROLLER COASTR | Multicenter; RA versus ELCA versus IVL (1:1:1) | *Angiography*: moderate calcifications were defined as radiopaque densities noted only with cardiac motion before a contrast injection (one side of the vessel or both), and severe calcifications were defined as radiopaque densities observed without cardiac motion | Percentage of stent expansion measured using OCT | ≥ 18 years old; diameter stenosis ≥ 70% or fractional flow reserve <0.8 / non-hyperemic indexes <0.89); RVD ≥ 2.5 and ≤ 4 mm; moderate or severe calcification estimated; stable coronary artery disease or non-ST-segment elevation acute coronary syndrome; culprit lesions at native vessels or coronary bypasses | Inability to tolerate DAPT; STEMI; Cardiogenic shock; impossibility to obtain informed consent; impossibility to conduct, at least, a 1-year follow-up |
| ROTA.Shock | Multicenter, open-lable; IVL versus RA (1:1) | *Angiography*: Severe calcification defined by coronary angiography as radiopacities noted without cardiac motion before contrast injection generally compromising both sides of the arterial lumen as assessed by the operator | MSA at the end of the procedure | Age > 18 years; written informed consent; severely calcified coronary lesion; clinically relevant coronary stenosis with an angiographic diameter stenosis > 50% and proof of ischemia; stable coronary artery disease or acute coronary syndromes; RVD between 2.5 and 4.0 mm | Cardiogenic shock; impaired renal function; bifurcation stenosis requiring a two-stent strategy; known allergy against protocol-required medications; history of bleeding diathesis or known coagulopathy; Women of childbearing age not taking effective contraceptives or breastfeeding women; chronic total occlusions; ISR |
| ROTA-CUT | Single-center; RA + CB vs RA (1:1) | *Angiography*: Severe calcification was defined as the presence of radiopacities noted without cardiac motion prior to contrast injection involving both sides of the arterial wall in at least one location and a total length of calcium (including segmented) of at least 15 mm extending partially into the target lesion area. Moderate calcium was defined as the presence of radiopacities only during the cardiac cycle before contrast injection with calcium extending partially into the target lesion | Postprocedural minimum stent area (in mm2) as assessed on the final post-procedure IVUS | Age ≥18 years of age; undergoing PCI for a de novo calcified lesion; RVD ≥2.5mm and ≤4.0mm; lesion length ≥5mm; moderate to severe calcification; eligible for PCI; willing and able to comply with all protocol-required follow-up evaluations | Cardiogenic shock; planned surgery (cardiac and non-cardiac); undergoing primary PCI for STEMI; pregnant, nursing, or is a woman of child-bearing potential; any other serious medical illness (e.g., cancer, end-stage congestive heart failure) that may reduce life expectancy to less than 12 months; currently participating in another investigational drug or device study; referred to coronary artery bypass grafting after heart team discussion |
| ROTAXUS | Multicenter; RA versus conventional balloon (1:1) | *Angiography*: Moderate to severe calcification of the target lesion angiographically graded as follows: none/mild; moderate (radiopacities noted only during the cardiac cycle before contrast injection); and severe (radiopacities noted without cardiac motion before contrast injection generally compromising both sides of the arterial lumen) | in-stent LLL (difference between the immediate post-procedure in-stent minimal lumen diameter and the in-stent minimal lumen diameter) at 9-month follow-up angiography | **Clinical:** Age > 18 years; proven coronary artery disease; angina II to IV following the Canadian Cardiovascular Society classification criteria and/or reproducible ischemia in the target area; informed written consent; **Angiographic:** de-novo lesion in a native coronary artery; RVD between 2.5 and 4.0 mm; luminal diameter reduction of 70% to 99%; moderate to severe calcification of the target lesion | **Clinical:** Myocardial infarction within 4 weeks; left ventricular ejection fraction <30%; limited long-term prognosis due to other conditions; **Angiographic:** Unprotected left main; bypass graft stenoses; ISR; chronic total occlusions; target vessel thrombus; target vessel dissection |
| Short-CUT^†^ | Multicenter; CB versus IVL (1:1) | *Angiography*: Radio-opacities involving both sides of arterial wall > 5mm at lesion site  *IVUS*: > 270° arc of Ca. > 5mm length Or 360° arc of Ca. | MSA at site of maximal calcification | Presentation with stable angina or NSTE-ACS with flat or down-trending biomarkers; de novo native lesion > 70% or 50-70% with evidence of hemodynamic significance; significant calcification | STEMI within 30 days; LVEF < 25%; cardiogenic shock or anticipated requirement for mechanical/pharmacological support; excessive tortuosity in vessel; evidence of dissection |
| SONAR^†^ | Multicenter; IVL versus RA (1:1) | *Angiography*: Moderate calcification is defined  as radiopacities noted only during the cardiac cycle before contrast dye injection, and severe calcification is defined as radiopacities seen without cardiac motion before contrast dye injection | Difference in the rate of peri-procedural MI (Type 4a of 4^th^ UDMI) | Age > 18 years old; stable or unstable angina pectoris, or a positive functional study for ischemia; eligible for PCI; consent prior to study inclusion; moderate to severe calcified lesion; the calcified lesion has a 50–90 % diameter stenosis with a RVD of 2.5–4.0 mm, and indication for PCI | Pregnant and/or breast-feeding; intend to have a major surgical intervention; experienced an acute MI 72 h prior to the index procedure; suffered a stroke or TIA or cerebrovascular accident; experienced a significant gastrointestinal or genitourinary bleed or has had any active bleeding within two months; planned revascularization; lesions not ideal for IVL or RA treatment; lesion longer than 40 mm; intraluminal thrombus; ISR |
| Tang et al | Single-center; CB versus conventional balloon (1:1) | *IVUS*: Calcium arc ≥ 180°, the calcium length ratio ≥ 0.5 | Death, MI, TVR, and composite MACE. | Native coronary artery with a stenosis of ≥ 70%; target lesion calcified with severely calcification | ISR; extremely tortuous or angulated lesions; dissection before balloon expansion; SVG; extremely narrow lesions in which IVUS could not get through |
| VICTORY^†^ | Multicenter; SHPB vs. IVL (1:1) | *Angiography*: Evidence of calcification at the lesion site by angiography (Grade 3), with fluoroscopic radio-opacities noted without cardiac motion prior to contrast injection involving both sides of the arterial wall AND/ OR Prior attempt at PCI & inability to expand balloon in target lesion  *OCT*: presence of ≥270°calcium | Final stent expansion (in percentage, %) assessed by OCT | **Clinical:** age ≥18 years; acute or chronic coronary artery disease with ischemia related symptoms and/or evidence of myocardial ischemia; angiographically-proven coronary artery disease; informed consent; **Angiographic:** single de novo target lesion stenosis of protected left main, or LAD, RCA or LCX with:  Stenosis of ≥70%;  Stenosis ≥50% and <70% with evidence of ischemia or IVUS minimum lumen area ≤4.0 mm²; RVD be ≥2.5 mm & ≤4.5mm; | STEMI or cardiogenic shock; any comorbidity or condition which may reduce compliance with this protocol; pregnant or nursing; unable to take a P2Y12 inhibitor; allergy to imaging contrast media; eGFR <30ml/min1.73m2; history of a stroke or TIA; active peptic ulcer or upper gastrointestinal bleeding;  untreated pre-procedural hemoglobin <10g/dL or intention to refuse blood transfusions if one should become necessary; allergy or intolerance to cobalt-chromium and/ or everolimus; life expectancy of less than 1 year. |
| Zou et al | Single-center; IVL versus conventional balloon (1:1) | *Angiography*: Clear, high-density shadows can be seen in the angiography, whether the heat is beating or not | Surgical success rate, intraoperative manipulation characteristics, procedural complication, and cumulative incidence of major adverse cardiovascular events | Not pregnant, aged 18–80 years; evidence of silent myocardial ischemia, stable or unstable angina, or earlier myocardial infarction; primary lesions and coronary artery lesions in situ as the target lesion; RVD of 2.5–4.0 mm and the length ≤ 40 mm; stenosis ≥ 70% or ≥ 50%, with evidence of ischemia; severe calcifications; TIMI grade 3 of the target vessel before the use of the test instrument; suitable for PCI treatment | Dissection in the target vessel; ISR; target lesion located in the ostium of the main vessels; left main coronary artery lesion or bypass graft lesion; tortuous and the test instrument was difficult to reach the target location or recover; history of active peptic ulcer or gastrointestinal hemorrhage; diagnosed with malignancy or with a life expectancy of less than 12 months; stroke within six months before enrollment in the study, excluding TIA and lacunar infarction; platelet counts < 80 × 109/L; severe liver and kidney function impairment; NYHA III or IV; allergic to heparin, contrast agents, aspirin, clopidogrel, or anesthetics; participating in clinical trials of other drugs or instruments; anticipated non-compliant |
|  | *^*^Data previously presented at the EuroPCR 2025 Conference.* ***^†^****Data previously presented at the TCT 2025 Conference.ACS: Acute Coronary Syndrome; ASS: Acetylsalicylic Acid; BMS: Bare Metal Stent; BRS: Bioresorbable Scaffold; CB: Cutting Balloon; CAG: Coronary Angiography; CT: Computed Tomography; DES: Drug-Eluting Stent; DAPT: Dual Antiplatelet Therapy; ECG: Electrocardiogram; ELCA: Excimer Laser Coronary Atherectomy; FFR: Fractional Flow Reserve; GFR: Glomerular Filtration Rate; ICF: Informed Consent Form; iFR: Instantaneous wave-Free Ratio; ISR: In-Stent Restenosis; IVI: Intravascular Imaging; IVL: Intravascular Lithotripsy; IVUS: Intravascular Ultrasound; LAD: Left Anterior Descending artery; LCx: Left Circumflex artery; LLL: Late Lumen Loss; LVEF: Left Ventricular Ejection Fraction; MACE: Major Adverse Cardiovascular Events; MB: Modified Balloon; MI: Myocardial Infarction; MSA: Minimum Stent Area; MS-CT: Multi-Slice Computed Tomography; NCB: Non-Compliant Balloon; NYHA: New York Heart Association; OCT: Optical Coherence Tomography; OA: Orbital Atherectomy; PAD: Peripheral Artery Disease; PCI: Percutaneous Coronary Intervention; PTCA: Percutaneous Transluminal Coronary Angioplasty; RA: Rotational Atherectomy; RCA: Right Coronary Artery; RVD: Reference Vessel Diameter; SB: Semi-Compliant Balloon; SHPB: Super High-pressure balloon; STEMI: ST-Elevation Myocardial Infarction; TIA: Transient Ischemic Attack; TIMI: Thrombolysis In Myocardial Infarction; SVG: Saphenous Vein Graft; TLR: Target Lesion Revascularization; TVF: Target Vessel Failure; TVR: Target Vessel Revascularization; UDMI: Universal Definition of MI; URL: Upper Reference Limit. Trial acronyms: BALI: Balloon Lithoplasty for Preparation of Severely Calcified Coronary Lesions, BASIL: Balloon Angioplasty versus Shockwave Intravascular Lithotripsy, COPS: Cutting Balloon to Optimize Predilatation for Stent Implantation, DIRO: direct comparison of RA vs OA for calcified lesions guided by OCT, ECLIPSE: Evaluation of Treatment Strategies for Severe CaLcIfic Coronary Arteries: Orbital Atherectomy vs. Conventional Angioplasty Technique Prior to Implantation of Drug-Eluting StEnts, EXIT-CALC: EXpansion of stents after Intravascular lithotripsy versus conventional predilatation in CALCified coronary arteries, ICARE: Intravascular Lithotripsy in Comparison to Rotational Atherectomy: An Evaluation by OFDI, ISAR-CALC: ComparIson of Strategies to PrepAre SeveRely CALCified Coronary Lesions, PREDICTION: A Prospective, Multicenter, Randomized Trial of New Cutting Balloon Versus Conventional Balloon for the Treatment of Severe Calcified Lesion, PREPARE-CALC: Comparison of Strategies to Prepare Severely Calcified Coronary Lesions, ROLLER COASTR-EPIC22: Rotational Atherectomy, Lithotripsy or Laser for the Treatment of Calcified Stenosis, ROTAXUS: Rotational Atherectomy Prior to Taxus Stent Treatment for Complex Native Coronary Artery Disease, Short-CUT: Shockwave Lithotripsy Compared to Cutting Balloon Treatment in Calcified Coronary Artery Disease, SONAR: ShOckwave ballooN or Atherectomy with Rotablation in calcified coronary artery lesions,* *VICTORY: Value of IVL Compared To OPN Non-Compliant Balloons for Treatment of RefractorY Coronary Lesions.* | | | | |

## Table S5. Summary of the CINeMA framework domains

| **Comparison** | **Within-study bias** | **Reporting bias** | **Indirectness** | **Imprecision** | **Heterogeneity** | **Incoherence** | **Confidence rating** |
| --- | --- | --- | --- | --- | --- | --- | --- |
| **ELCA:IVL** | No concerns | Low risk | No concerns | No concerns | Some concerns | No concerns | Moderate |
| **ELCA:RA** | No concerns | Low risk | No concerns | No concerns | Some concerns | No concerns | Moderate |
| **IVL:MB** | Some concerns | Low risk | No concerns | No concerns | No concerns | No concerns | Moderate |
| **IVL:NCB** | Some concerns | Low risk | No concerns | No concerns | Some concerns | No concerns | Moderate |
| **IVL:SHPB** | Major concerns | Low risk | No concerns | No concerns | No concerns | No concerns | Low |
| **IVL:RA** | Some concerns | Low risk | No concerns | No concerns | No concerns | No concerns | Moderate |
| **MB:NCB** | Some concerns | Low risk | No concerns | No concerns | Some concerns | No concerns | Moderate |
| **MB:SHPB** | Some concerns | Low risk | No concerns | No concerns | No concerns | No concerns | Moderate |
| **NCB:OA** | No concerns | Low risk | No concerns | No concerns | No concerns | No concerns | High |
| **OA:RA** | Some concerns | Low risk | No concerns | No concerns | Some concerns | No concerns | Moderate |
| **RA:RA + CB** | Major concerns | Low risk | No concerns | No concerns | No concerns | Major concerns | Low |
| **ELCA:MB** | Some concerns | Low risk | No concerns | No concerns | Some concerns | Major concerns | Low |
| **ELCA:NCB** | No concerns | Low risk | No concerns | Some concerns | No concerns | Major concerns | Low |
| **ELCA:OA** | No concerns | Low risk | No concerns | No concerns | Some concerns | Major concerns | Low |
| **ELCA:SHPB** | Some concerns | Low risk | No concerns | Some concerns | No concerns | Major concerns | Low |
| **ELCA:RA + CB** | Some concerns | Low risk | No concerns | Some concerns | No concerns | Major concerns | Low |
| **IVL:OA** | Some concerns | Low risk | No concerns | No concerns | Some concerns | Major concerns | Low |
| **IVL:RA + CB** | Some concerns | Low risk | No concerns | No concerns | Some concerns | Major concerns | Low |
| **MB:OA** | Some concerns | Low risk | No concerns | No concerns | Some concerns | Major concerns | Low |
| **MB:RA** | Some concerns | Low risk | No concerns | No concerns | No concerns | Major concerns | Low |
| **MB:RA + CB** | Some concerns | Low risk | No concerns | No concerns | Some concerns | Major concerns | Low |
| **NCB:SHPB** | Some concerns | Low risk | No concerns | No concerns | Some concerns | Major concerns | Low |
| **NCB:RA** | Some concerns | Low risk | No concerns | No concerns | Some concerns | Major concerns | Low |
| **NCB:RA + CB** | Some concerns | Low risk | No concerns | No concerns | No concerns | Major concerns | Low |
| **OA:SHPB** | Some concerns | Low risk | No concerns | No concerns | Some concerns | Major concerns | Low |
| **OA:RA + CB** | Some concerns | Low risk | No concerns | No concerns | Some concerns | Major concerns | Low |
| **SHPB:RA** | Some concerns | Low risk | No concerns | No concerns | No concerns | Major concerns | Low |
| **SHPB:RA + CB** | Some concerns | Low risk | No concerns | No concerns | Some concerns | Major concerns | Low |
| *CB: Cutting balloon; CINeMA: Confidence In Network Meta-Analysis; ELCA: Excimer Laser Coronary Angioplasty; IVI: Intravascular Imaging; IVL: Intravascular Lithotripsy; MB: Modified balloon; NCB: Non-compliant balloon; OA: Orbital Atherectomy; SHPB: Super High-pressure balloons; RA: Rotational Atherectomy* | | | | | | | |

## Table S6. Additional trials characteristics for transitivity evaluation

| **Trial** | **Inclusion of ACS and (%)** | **LAD PCI ≥50%** | **Inclusion of severe calcification only** | **Inclusion of calcifications defined by IVI** | **IVI Type** | **IVI pre-PCI** | **Uncrossable lesions inclusion allowed** |
| --- | --- | --- | --- | --- | --- | --- | --- |
| BALI | X | X | X | X | OCT | X | X |
| BASIL | X |  | X | X | IVUS | X |  |
| COPS | X | X | X | X | IVUS | X |  |
| DIRO |  | X |  | X | OCT | X | X |
| ECLIPSE | X | X | X | X | IVUS/OCT | X |  |
| Elyamani et al |  | X | X |  |  |  | X |
| EXIT-CALC |  | X | X | X | OCT | X |  |
| Han et al |  | X | X | X | IVUS | X | X |
| ICARE |  | X |  |  | OCT | X |  |
| ISAR-CALC |  | X |  |  | OCT |  |  |
| Li et al | X | X | X | X | IVUS | X | X |
| PREDICTION | X |  | X |  | IVUS | X |  |
| PREPARE CALC | X | X | X |  |  |  | X |
| ROLLER COASTR | X | X |  |  | OCT | X | X |
| ROTA-Shock | X |  | X |  | OCT | X | X |
| ROTA-CUT |  | X |  |  | IVUS | X | X |
| ROTAXUS | X | X |  |  |  |  | X |
| Short-CUT | X |  | X | X | IVUS | X | X |
| SONAR | X | X |  |  | IVUS/OCT | X |  |
| Tang et al | X | X | X | X | IVUS | X | X |
| VICTORY |  |  | X | X | OCT | X | X |
| Zou et al |  | X | X |  |  |  | X |
| Baseline, procedural and definitions included in Table 1 and in Table S3 and S4 are not reported in this table to avoid redundancy | | | | | | | |

## Table S7. Sensitivity Analysis-Based Rankogram (P-Score) for the Primary Outcome

| High RoB excluded | |  | Ca. IVI-defined | |
| --- | --- | --- | --- | --- |
| SHPB | 0.0534 |  | RA + CB | 0.2225 |
| RA + CB | 0.1433 |  | IVL | 0.3190 |
| MB | 0.2386 |  | MB | 0.3335 |
| RA | 0.4939 |  | SHPB | 0.4687 |
| IVL | 0.5412 |  | RA | 0.5352 |
| OA | 0.7726 |  | OA | 0.7340 |
| ELCA | 0.7865 |  | NCB | 0.8871 |
| NCB | 0.9704 |  |  |  |
| *P-score of 1 indicates the worst performance and 0 the best. Ca.: Calcification; CB: Cutting balloon; ELCA: Excimer Laser Coronary Angioplasty; IVI: Intravascular Imaging; IVL: Intravascular Lithotripsy; MB: Modified balloon; NCB: Non-compliant Balloon; OA: Orbital Atherectomy; SHPB: Super High-pressure balloons; RA: Rotational Atherectomy; RoB: Risk of Bias.* | | | | |

## Table S8. Rankogram analyses (P-Score) for secondary clinical and procedural outcomes

| Death | |  | MI | |  | ST | |  | Repeat revascularization | |  | Perforation | |  | Proc. Success^*^ | |
| --- | --- | --- | --- | --- | --- | --- | --- | --- | --- | --- | --- | --- | --- | --- | --- | --- |
| SHPB | 0.6466 |  | ELCA | 0.7685 |  | SHPB | 0.7418 |  | RA | 0.7141 |  | SHPB | 0.7930 |  | RA + CB | 0.1249 |
| RA + CB | 0.6324 |  | SHPB | 0.7033 |  | RA + CB | 0.6436 |  | OA | 0.5804 |  | NCB | 0.7078 |  | RA | 0.1384 |
| MB | 0.5949 |  | IVL | 0.5108 |  | NCB | 0.6315 |  | RA + CB | 0.5555 |  | IVL | 0.5695 |  | SHPB | 0.2056 |
| IVL | 0.5226 |  | NCB | 0.4832 |  | IVL | 0.5376 |  | ELCA | 0.5408 |  | MB | 0.5387 |  | IVL | 0.4956 |
| NCB | 0.4913 |  | RA | 0.4504 |  | MB | 0.5129 |  | NCB | 0.5262 |  | RA + CB | 0.4072 |  | NCB | 0.7136 |
| RA | 0.4194 |  | RA + CB | 0.4368 |  | ELCA | 0.3158 |  | IVL | 0.4382 |  | OA | 0.3449 |  | OA | 0.7302 |
| OA | 0.3557 |  | OA | 0.4005 |  | RA | 0.3091 |  | MB | 0.3416 |  | RA | 0.3297 |  | MB | 0.7909 |
| ELCA | 0.3373 |  | MB | 0.2465 |  | OA | 0.3076 |  | SHPB | 0.3032 |  | ELCA | 0.3093 |  | ELCA | 0.8009 |
| *^*^For procedural success, a P-score of 1 indicates the worst performance and 0 the best; for all other outcomes, a P-score of 1 indicates the best performance and 0 the worst. Ca.: Calcification; CB: Cutting balloon; DS: Diameter Stenosis; ELCA: Excimer Laser Coronary Angioplasty; IVI: Intravascular Imaging; IVL: Intravascular Lithotripsy; MB: Modified balloon; MI: Myocardial Infarction; NCB: Non-compliant balloon; OA: Orbital Atherectomy; SHPB: Super High-pressure balloons; RA: Rotational Atherectomy; ST: Stent Thrombosis.* | | | | | | | | | | | | | | | | |

# Supplemental Figures

## Figure S1. PRISMA 2020 flow chart for study selection

^a^Conference abstracts and presentations were identified by manually screening the online programs of major cardiology meetings (including Transcatheter Cardiovascular Therapeutics, EuroPCR, and European Society of Cardiology Congress) from the most recent year within the predefined search period. These records were not retrieved through database indexing but through a targeted manual search.

## Figure S2. Risk-of-Bias Assessment with Cochrane RoB tool v2 for randomized trials

## Figure S3. Funnel plot distribution of mean differences in minimal stent area for each calcium modification device comparison

CB: Cutting Ballons; ELCA: Excimer Laser Coronary Angioplasty; IVL: Intravascular Lithotripsy; MB: Modified Balloons; NCB: Non-compliant Balloons; OA: Orbital Atherectomy; SHPB: Super High-pressure balloons; RA: Rotational Atherectomy.

## Figure S4. Sensitivity analysis of the primary outcome excluding High Risk of Bias Studies

CB: Cutting Balloons; CI: Confidence Interval; ELCA: Excimer Laser Coronary Angioplasty; IVL: Intravascular Lithotripsy; MB: Modified Balloons; MD: Mean Difference; NCB: Non-Compliant Balloon; OA: Orbital Atherectomy; SHPB: Super High-pressure balloons; RA: Rotational Atherectomy.

## Figure S5. Sensitivity analysis of the primary outcome including only studies using an IVI-based definition of severe calcification

CB: Cutting Balloons; CI: Confidence Interval; IVI: Intravascular Imaging; IVL: Intravascular Lithotripsy; MB: Modified Balloons; MD: Mean Difference; NCB: Non-Compliant Balloon; OA: Orbital Atherectomy; RA: Rotational Atherectomy; SHPB: Super High-pressure balloons.

## Figure S6. Sensitivity analysis of the primary outcome excluding the ECLIPSE trial

Results are expressed as Risk Ratios and 95% Confidence Intervals. For MSA, a P-score of 1 indicates the worst performance and 0 the best. CB: Cutting Balloon; ECLIPSE: Evaluation of Treatment Strategies for Severe Calcific Coronary Arteries: Orbital Atherectomy vs Conventional Angioplasty Technique Prior to Implantation of Drug-Eluting Stents; ELCA: Excimer Laser Coronary Angioplasty; IVL: Intravascular Lithotripsy; MB: Modified Balloons; MSA: Minimal Stent Area; NCB: Non-compliant Balloons; OA: Orbital Atherectomy; SHPB: Super High-pressure balloons; RA: Rotational Atherectomy.

## Figure S7. Post-PCI diameter stenosis Network analysis

CB: Cutting Balloons; CI: Confidence Interval; ELCA: Excimer Laser Coronary Angioplasty; IVL: Intravascular Lithotripsy; MB: Modified Balloons; MD: Mean Difference; NCB: Non-Compliant Balloon; OA: Orbital Atherectomy; SHPB: Super High-pressure balloons; PCI: Percutaneous Coronary Intervention; RA: Rotational Atherectomy.

## Figure S8. League tables for secondary clinical outcomes

Results are expressed as Risk Ratios and 95% Confidence Intervals. CB: Cutting Balloon; ELCA: Excimer Laser Coronary Angioplasty; IVL: Intravascular Lithotripsy; MB: Modified Balloons; NCB: Non-compliant Balloons; OA: Orbital Atherectomy; SHPB: Super High-pressure balloons; RA: Rotational Atherectomy.

## Figure S9. League tables for secondary procedural outcomes

A trend toward fewer vessel perforations with SHPB is highlighted in light green, while statistically significant differences favoring SHPB, RA and RA combined with CB for procedural success are indicated in dark green. Results are expressed as Risk Ratios and 95% Confidence Intervals. CB: Cutting Balloon; ELCA: Excimer Laser Coronary Angioplasty; IVL: Intravascular Lithotripsy; MB: Modified Balloons; NCB: Non-compliant Balloons; OA: Orbital Atherectomy; SHPB: Super High-pressure balloons; PCI: Percutaneous Coronary Intervention; RA: Rotational Atherectomy.
